# Is There a Positive Side to T Cell Exhaustion?

**DOI:** 10.3389/fimmu.2019.00111

**Published:** 2019-01-29

**Authors:** Graham Pawelec

**Affiliations:** ^1^Second Department of Internal Medicine, University of Tübingen, Tübingen, Germany; ^2^Cancer Solutions Program, Health Sciences North Research Institute, Sudbury, ON, Canada

**Keywords:** T cell exhaustion, cell senescence, organ transplantation, cancer immunity, cancer immunotherapy, Hayflick limit, immunosurveillance

## Abstract

T cell “exhaustion” describes a state of late-stage differentiation usually associated with active prevention of functionality via ligation of negative signaling receptors on the cell surface, and which can be reversed by blocking these interactions. This contrasts with T cell “senescence,” which has been defined as a state that is maintained by intrinsic internal cell signaling (caused by DNA damage or other stresses) and which can be reversed pharmacologically. Interventions to alleviate these two different categories of inhibitory pathways may be desirable in immunotherapy for cancer and possibly certain infectious diseases, but reciprocally inducing and maintaining these states, or some properties thereof, may be beneficial in organ transplantation and autoimmunity. Even under physiological non-pathological conditions, T cell exhaustion and senescence may play a role in the retention of T cell clones required for immunosurveillance, and prevent their loss via elimination at the Hayflick limit. This essay briefly reviews T cell exhaustion in contrast to replicative senescence, and circumstances under which their modulation may be beneficial.

## Introduction

In common parlance, the term exhaustion has solely negative connotations, deriving from the Latin meaning “completely emptied out.” However, an entity in a state of exhaustion can recoup and recover; exhaustion prevents further use of resources and allows potential regeneration. Used in the context of T cell immunity, the term appears first to have been applied 20 years ago to what remains the most thoroughly investigated model system, chronic infection with a certain strain of LCMV (1), although in the context of chronic exposure to tumors, it was already suggested a decade earlier (2). Moreover, the latter authors demonstrated that tumor rejection in young mice was compromised in older individuals, suggested that exhaustion might be the reason for this, and documenting potential reversibility of this state (2). As many patients with solid cancers are elderly, there is a concern that, as in old mice, their ability to reject tumors is compromised by T-cell exhaustion. Defining and reversing or preventing this state is then considered crucial for treating these patients. Currently, there is a great deal of interest in T cell dysfunction in human cancer and its potential reversibility by administering immunomodulatory antibodies, particularly anti-PD-1 or anti-PD-L1 at the present time. Although generally viewed in pathological terms, and commonly conflated with “senescence” in the literature (3), a state of reversible exhaustion could be viewed as a physiological mechanism facilitating the retention of antigen-specific T cells in the repertoire under chronic stimulation. The expression of PD-1 by the T cell is reported to be one of the hallmarks of exhaustion (4), but is clearly not an exclusive marker thereof (analogous to the downregulation of CD27 and CD28 by activated and differentiating cells taken as indicative of T cell senescence but not specific markers thereof). The interaction between the negative signaling receptor PD-1 on the T cell and PD-L1 or PD-L2 on the antigen-presenting cell is one of several physiological feedback control mechanisms which serve to block T cell proliferation under chronic antigenic stimulation. T cells, like other somatic tissues, are unable to divide indefinitely and eventually reach the equivalent of the “Hayflick Limit” first described in fibroblasts over half a century ago (5). However, unlike human fibroblasts, where telomere attrition triggers growth arrest (6), a state of “replicative senescence” is rarely or never arrived at by human T cells in long-term culture because unlike most somatic cells, T cells can activate telomerase (7). Rather, clonal analysis suggests gradual attrition of the population by increasing fractions of daughter cells undergoing activation-induced cell death by apoptosis until the clone is lost. There is a great deal of clonal heterogeneity in this respect, with final loss of the longest-lived clone at over 70 cumulative populations doublings (CPD), but with an average clonal “life expectancy” of only around 30 PD (8). Thus, to retain T cell clones within the repertoire, a means of preventing them from reaching the Hayflick Limit while retaining potential specific function would be advantageous. Induction of a reversible state of exhaustion rather than replicative senescence, which is commonly believed to be permanent under normal physiological circumstances, or cell death, which is certainly permanent, would be one way of achieving this. There is some evidence that clonal attrition of T cells [many specific for the common persistent herpesvirus Cytomegalovirus (CMV)] does occur, and has implications for survival, but this has only been reported at the very end of life (9). This may reflect the final breakdown of essential anti-viral surveillance after over 80 years of chronic exposure, and illustrates the importance of retaining such T cells in the repertoire and the very efficient but not perfect mechanisms involved in their retention. Be that as it may, it remains important clearly to distinguish between exhaustion and senescence and to determine means of identifying these states in human T cells and whether there may be circumstances under which one or other of these states is advantageous to the individual. A corollary of this notion is that interventions to alter these states either with immunomodulatory antibodies (“checkpoint blockade,” for which the 2018 Nobel Prize in Physiology or Medicine was awarded, see https://www.nobelprize.org/prizes/medicine/2018/prize-announcement/) or with pharmacological agents (10) would need to be carefully selected according to the circumstances. This could be important in the context of attempts to reverse exhaustion (4) or to eliminate senescent cells (11) as opposed to “rejuvenating” the latter (10).

## Hallmarks of Replicative Senescence

The classic hallmark features of replicative senescence as defined in cultured fibroblasts is telomere shortening with each cell division until DNA damage repair pathways are triggered to prevent further cell division (12). Alternatively, DNA damage directly triggering these mechanisms results in a similar state of blocked proliferation (13). For still-unknown reasons, such cells often express so-called “senescence-associated beta-galactosidase” (14), which remains the most reliable marker of senescent cells. Characteristically, such cells express cell cycle control proteins such as p16, p21, p27, p53 which lock the cell into G1 (15); these factors are often referred to as markers of senescence but are clearly not in themselves markers for that state, as are DNA damage foci. Such physiological mechanisms act as tumor suppressors and should result in allowing time for the cell to repair DNA (16). If this fails, apoptosis should take place, but in somatic cells with short telomeres that lack telomerase DNA repair cannot occur, and therefore such cells persist because for unclear reasons they become apoptosis-resistant (17). A fail-safe mechanism requiring the elimination of senescent cells by the immune system (18) may also become less effective with age (19, 20). This results in the accumulation of senescent cells with increasing age, which was suggested to have deleterious consequences for the organism (16). A major reason for this is thought to be that senescent cells secrete a variety of generally pro-inflammatory factors known as the “senescence-associated secretory phenotype” or SASP (21) which have a deleterious systemic effect on multiple organs and contribute to “inflammaging” (22). It has been shown that the selective elimination of transplanted senescent cells conveys health benefits and potentially extend lifespan in recently developed animal models (23). It therefore appears that there are likely to be clear negative health consequences for possessing quantities of senescent cells that accumulate with age. The question of interest in this essay is whether and to what extent these findings, mostly limited to fibroblasts and keratinocytes, also apply to T cells?

## Hallmarks of T Cell Senescence

The terms T cell “exhaustion” and “senescence” are often applied loosely and interchangeably in the literature, and this has resulted in some degree of confusion, compounded by failure to distinguish differences between CD4^+^ and CD8^+^ T cells and possible species differences (24). Following the example of the work on non-lymphoid cell types, let us first consider T cell senescence in cell culture models. The earliest approaches cultured peripheral blood mononuclear cells *in vitro* with mitogens and the growth factor interleukin 2 (25). Under these conditions, mostly CD8^+^ T cells predominate in the population, which ceases to proliferate after a low number of cumulative population doublings (CPD), with shortened telomeres. Studies showed that downregulation of the costimulatory receptor CD28 on the T cell surface correlated with waning proliferation, due to the requirement for intermittent restimulation via the T cell receptor for antigen together with a second signal delivered by ligation of CD28 by CD80 or CD86 on the antigen-presenting cell surface; this signal was also required for telomerase upregulation. In culture, such cells became apoptosis-resistant (26). Consistent with this, long-term culture of CD4^+^ T cells also resulted in gradual downregulation of CD28 expression, although this was associated with an increased, not decreased, susceptibility to apoptosis (27). The difference between CD4^+^ and CD8^+^ T cell cultures may reflect different requirements for maintaining viability in these subsets in that type I interferons were reported to enable CD4^+^ T cell survival, albeit perhaps at the cost of contributing to inflammaging (28). At that time, our own search for senescence markers in CD4^+^ T cell clones identified rather few in addition to CD28 that changed robustly with increasing CPD in culture. These included other costimulatory receptors CD134 and CD154 but with a great deal of inter-clonal heterogeneity (29). Even for CD28 expression, certain clones re-expressed CD28 with increasing culture time, which we correlated with a decreased ability of the clones to secrete TNF. This is consistent with a report that TNF downregulates CD28 expression (30) and with our observations that TNF can directly inhibit some clones (31). These findings serve to illustrate the heterogeneity of *in vitro* T cell “aging models” at the clonal level, reflected also in their uninformative expression of “senescence” markers p 16, p21, and p27 (32) and variable capacity to maintain or even increase telomere lengths. The usefulness of senescence-associated beta-galactosidase expression in T cells is also unclear. Thus, disentangling differentiation stages in human T cells in order to distinguish late-differentiated cells from senescent cells remains a challenge, both *in vitro* and *in vivo*, as there are no hard and fast markers of senescence. For CD8^+^ T cells, an extended phenotype of the TEMRA stage (“T effector memory cell re-expressing CD45RA”) is the most likely to define a population that at least contains senescent cells, even if not all cells with this phenotype are indeed senescent. Typing for cells that are CD8^+^CD27^−^CD28^−^CD57^+^ and KLRG-1^+^ will allow detection of these cells, which do bear some resemblances to replicatively senescent fibroblasts, i.e., short telomeres, lack of proliferation, defective mitochondrial function, higher p53, oxidative damage and higher levels of reactive oxygen species (ROS) as well as higher p38 mitogen-activated protein kinase (p38 MAPK) (33). They are also capable of secreting high levels of pro-inflammatory mediators reminiscent of the fibroblast SASP, including TNF and other factors (34). Expression of KLRG-1 delivers negative regulatory signals to the T cell when ligated by E-cadherin (35), and whereas the presence of CD57 is associated with poor or absent proliferative capacity, the mechanism for this effect in unknown. Notwithstanding similarities to replicatively senescent fibroblasts, such T cells differ in that they can still be stimulated to proliferate (36). Hence one must question whether their arrested state represents true replicative senescence or whether their further extensive expansion is physiologically controlled to maintain essential functionality (e g., anti-CMV immunosurveillance) by a negative feedback mechanism which might prevent their reaching the Hayflick limit and being lost from the system by clonal attrition. As mentioned above, there are some limited data supporting the notion that such clonal attrition of CD8^+^ T cells does occur at the very end of life in the oldest old, and is associated with their mortality (9). From this point of view, CD8^+^ T cells considered senescent on the basis of a CD8^+^CD27^−^CD28^−^CD57^+^ and KLRG-1^+^ phenotype are still performing ongoing essential immune functions but are blocked from further replication unless pharmacologically manipulated, for example, by inhibiting p38 MAPK signaling which results in increased proliferation, telomerase activity and mitochondrial biogenesis (37). Studies *ex vivo* on freshly-isolated CD4^+^ T cells (selected for double CD27- and CD28-negativity as surrogate senescence markers) have more recently further dissected the physiological state of these cells. This work showed that p38 MAPK can be intrinsically activated by intracellular stress signaling, for example as a result of DNA damage or ROS activation of the AMP-activated protein kinase (AMPK) pathway (38). It was argued that senescence is an active state maintained by Erk, Jnk, and P38 MAPK signaling, all three of which were regulated by sestrins, and that pharmacological inhibition thereof rejuvenated these CD4 cells (10). Abrogation of such control mechanisms might contribute to disease and tumorigenesis but conceivably controlled short-term application could result in beneficial effects, as demonstrated by the improvement of some characteristics of the anti-influenza vaccine response in old mice (10).

## What Is T Cell Exhaustion?

As alluded to in the Introduction, a state of exhaustion is defined by reduced functionality which can be recovered by manipulating extrinsic regulatory pathways, for example, by checkpoint blockade. Such exhausted cells are physiologically intact, and are commonly found in situations of chronic infections and cancer where chronic antigenic stimulation from a source that cannot be cleared prevents many of the responding T cells from reverting to quiescent memory cells. As also discussed above, if responding cells continued to proliferate, they would eventually reach their Hayflick limit and become replicatively senescent or undergo clonal deletion (39). This can be prevented by some of the mechanisms discussed above, but alternatively, before this state is reached, the impact of heightened inflammatory status (i.e., a certain cytokine/chemokine milieu (40) together with chronic antigen exposure) may render the cells exhausted, especially in the absence of CD4^+^ T cell help (41). This process proceeds in an ordered manner, at least in murine LCMV models, whereby it may be challenging to distinguish between T cell differentiation pathways and events associated with exhaustion. Thus, IL 2 production and proliferative capacity decrease first, but this also happens during differentiation of naïve T cells to effector cells. However, as exhaustion progresses, cytolytic activity is lost and cytokine secretion decreased (4), rather than increased as in replicative senescence via the SASP. This leads to physiological alterations to responding T cells prior to their reaching the Hayflick limit, which can be modeled *in vitro* in long-term cultured T cell clones (42). Exhausted T cells do not necessarily possess short telomeres or manifest DNA damage, but they nonetheless express a range of negative receptors that act to dampen their responses. These include an ever-increasing number of surface molecules in different systems and species, such as PD-1 (CD279) (43), CTLA-4 (CD152) (44), TIM3 (45), LAG3 (46), TIGIT (47), 2B4 (CD244) (48), CD160 (BY55) (49), etc. as well as transcription factors such as Eomes (50). A central role for the IL 12 family member IL 27 has been proposed, at least in mice, because it is a major driver of IL 10 production and, most importantly, was recently found to act as a master controller of an entire constellation of negative regulatory surface receptors, not only including PD-1, TIM3, LAG3, and TIGIT but also two novel receptors, PROCR and PDPN (51). Nonetheless, it should not be forgotten that these negative receptors are all components of normal physiological immune response control mechanisms and their presence does not necessarily mark exhaustion (or senescence); rather it is context-dependent (52).

Recent advances in analytical tools have enable unprecedentedly detailed characterization of exhausted T cells in humans, mostly in the context of HIV infection and cancer. In both indications, the use of antibodies to block interactions between negative receptors and their ligands has shown reversibility of exhaustion, most dramatically currently in cancer immunotherapy. Single cell transcriptomic, epigenetic and deep phenotyping approaches using mass cytometry profiling are revealing distinct heterogeneous clusters of exhausted T cells, enabling “exhaustion severity” algorithms to be identified. These were shown to be shared between healthy donors, HIV patients and cancer, but quantitatively different in either disease, related to disease severity, and dynamic according to therapeutic intervention, at least for HIV therapy. Hence, combinations of markers for individualizing immunotherapy regimens specific for the different exhausted T cell profiles could be defined (53). In this manner, some progress should be possible to bridge the divide between correlations and causation by close monitoring of patients on therapy. This will be extremely important, especially in the field of cancer therapy, where major successes have been achieved first using anti-CTLA-4 antibodies and more recently anti-PD-1 antibodies or antibodies to one of its ligands, PD-L1. A demonstration of the crucial importance of the ability of anti-PD-1 antibodies to reinvigorate T cell responses was recently demonstrated by the finding that melanoma patients failing to respond to immunotherapy simply had too high a tumor burden, such that this therapy alone might be successful in all patients provided that the bulk of residual disease did not exceed a certain limit (54). Consistent with this, these authors also showed that, as expected in systems with high redundancy, it may not be so easy to overcome the established momentum of the feedback circuits. Thus, in the classic case of blocking PD-1 to reinvigorate exhausted CD8^+^ T cells, effects may be short-lived and cells rapidly become exhausted again in the continued presence of high antigen concentrations. The epigenetic profile of reinvigorated exhausted cells (55) was reported to remain different from non-exhausted effector and memory cells, suggesting an epigenetic profile resistant to readjustment by merely blocking PD-1 signaling (56). Given the range of other negative receptors expressed by exhausted cells, this would not be unexpected and emphasizes the emerging consensus that combinatorial treatments will be necessary for cancer and other immunotherapies (57). Nonetheless, manipulation of early epigenetic programming might already be sufficient to alleviate many of these negative effects of exhaustion (58). Thus, as evidence accumulates for the negative effects of T cell exhaustion on the outcome of cancer immunotherapy much ongoing effort is directed toward identifying targetable pathways and surface receptors other than CTLA-4 and PD-1, currently the only two targets for which there are licensed immunomodulatory antibodies available. Blocking the interactions of these separate from or in addition to CTLA-4 and PD-1 might be expected to increase the potential to abrogate T cell exhaustion and reveal anti-cancer immune function. There is currently much interest in TIGIT (59) and multiple other targets in this context, as well as targeting events and pathways downstream of receptor signaling. Metabolic reprogramming associated with exhaustion involves the upregulation of many anabolic pathways (60). This suggests that manipulating glycolytic and mitochondrial metabolism might underlie the action of multiple different negative receptors associated with exhaustion.

## Is T Cell Exhaustion Ever Demonstrably Beneficial?

Notwithstanding the predominantly negative effects of T cell exhaustion in cancer and infectious disease, there may be situations where decreasing immunity is desirable, for example in organ transplantation and especially in autoimmunity. This area is less-well investigated than in cancer and infection, but there is some evidence that similar processes could be exploited for controlling autoimmunity. Thus, for example, transcriptional signatures characteristic of CD8+ T-cells were reported to be associated with better prognosis in several different autoimmune diseases including type 1 diabetes, anti-neutrophil cytoplasmic antibody-associated vasculitis, systemic lupus erythematosus, idiopathic pulmonary fibrosis and dengue haemorrhagic fever (61). Hence, it was concluded that T-cell exhaustion is a double-edged sword with a central role for outcome of both autoimmune disease and infection and cancer.

In the context of transplantation, in adoptive immunotherapy for myeloid leukemias, it was reported that persistence of potentially exhausted recipient CD8^+^ T cells (PD-1^+^Tim-3^+^, with high T-bet and Eomes expression) correlated with better therapeutic effect (62). A current trial (NCT02533180) is evaluating whether the establishment of an exhausted phenotype after withdrawal of immunosuppression following liver transplantation is responsible for maintaining tolerance (63). In allografting, induction of state reminiscent of CD8^+^ T cell exhaustion when a kidney from a CMV-seropositive donor was transplanted into a CMV-negative recipient may be associated with a beneficial effect (64). In a murine model, a substantially greater T cell exhaustion status in cells from recipients of transplanted hearts tolerating rather than rejecting the allograft suggests a contribution to the prevention of rejection of the exhausted state (65). Fuller consideration of the role and potential exploitation of T cell exhaustion is to be found in recent reviews (66, 67).

## Conclusions

Exhausted T cells are found under different circumstances of chronic antigenic stimulation and are characterized by reduced but recoverable functionality, actively maintained by signaling through negative costimulatory interactions. These cells are biologically intact but subject to physiological negative regulatory control. CD8^+^ T cells with characteristics reminiscent of replicative senescence in fibroblasts may also be present under similar conditions, although CD4^+^ T cells are more likely to be clonally deleted after many cell divisions, due to increased susceptibility to apoptosis. This may contribute to the observed age-associated inversion of the CD4:8 cell ratio in some older adults, facilitating the accumulation of late-stage differentiated CD8 cells, which reflects an “immune risk profile” associated with shorter remaining lifespan in longitudinal studies (68). In contrast, a high CD4:8 ratio caused by the accumulation of CD4^+^ T cells that could be maintained by the recently-defined sestrin-dependent Erk/Jnk/p38 MAPK pathway (10), may also associate with a shorter remaining lifespan (69). Rather than viewing CD8^+^ T cell exhaustion entirely negatively, it is argued that this physiological process may exist to control and extend the immunological reserve required for maintaining surveillance of chronic infections. This is characterized by the maintenance of cellular integrity, and is controlled by extrinsic immunological regulatory mechanisms, in contrast to senescent cells which are controlled intrinsically by cellular stress responses.

## Author Contributions

The author confirms being the sole contributor of this work and has approved it for publication.

### Conflict of Interest Statement

The author declares that the research was conducted in the absence of any commercial or financial relationships that could be construed as a potential conflict of interest.
